# Trend in young coronary artery disease in China from 2010 to 2014: a retrospective study of young patients ≤ 45

**DOI:** 10.1186/s12872-016-0458-1

**Published:** 2017-01-07

**Authors:** Xin Wang, Ming Gao, Shanshan Zhou, Jinwen Wang, Fang Liu, Feng Tian, Jing Jin, Qiang Ma, Xiaodi Xue, Jie Liu, Yuqi Liu, Yundai Chen

**Affiliations:** 1 Department of Health Statistics, School of Public Health, Fourth Military Medical University, Xi’an, Shanxi Province China; 2 Center For Disease Control And Prevention Of The PAP, Beijing, China; 3 Department of Cardiology, PLA General Hospital, Beijing, 100853 China; 4 ICU of The first phase Beijing Tsinghua Changgeng Hospital, Beijing, 100044 China; 5 AnZhen Hospital, Capital Medical University, Beijing, 100029 China; 6 Department of Cardiology of AnZhen Hospital, Beijing, 100029 China

**Keywords:** Young, Coronary heart disease, Risk factors, Prevention, Prognosis

## Abstract

**Background:**

The incidence of young coronary heart disease (CHD, ≤45 years) in China is increasing. Secondary prevention to counter this trend is an important contemporary public health issure.

**Methods:**

A total of 5288 patients (≤45 years) diagnosed with CHD and hospitalized at the Chinese PLA General Hospital and Anzhen Hospital, both in Beijing, were enrolled after satisfying the inclusion criteria.

**Results:**

Young CHD patients increased in number from 2010 to 2014, especially men. Among the studied patients, there was no significant change over those years in blood pressure, but heart rate increased significantly (*P* < 0.05) and body mass index showed a rising trend (*P* > 0.05). The incidence of hypertension increased from 40.7 to 47.5%, diabetes from 20.3 to 26.1%, and hyperlipidemia from 27.3 to 35.7% (*P* < 0.05). However, the incidences of smoking and drinking both trended downward (*P* < 0.05). The levels of total cholesterol and triglycerides also showed a downward trend (*P* < 0.05), as did levels of low-density lipoprotein, but not to the point of statistical significance (*P* > 0.05). Mortality during hospitalization decreased significantly from 2010 to 2014 (*P* < 0.05), but there was no significant improvement in the incidences of cardiac death and major adverse cardiovascular events (MACE) after 1-year follow-up (*P* > 0.05).

**Conclusions:**

Over the 5 years studied, the overall incidence of cardiac death and MACE for young CHD patients (≤45 years) has shown little improvement. Secondary prevention of young CHD, and its risk factors, as well as appropriate courses of medical treatment must be further elucidated.

**Electronic supplementary material:**

The online version of this article (doi:10.1186/s12872-016-0458-1) contains supplementary material, which is available to authorized users.

## Background

As economy and standard of living improve, the incidence of coronary heart disease (CHD) rises [1]. This is a major public health concern, as the overall mortality from acute myocardial infarction (AMI) also increases. The results of the China Patient-centered Evaluation Assessment of Cardiac Events (ChinaPEACE) showed that, from 2001 to 2011, the hospitalization rate for ST-segment elevation myocardial infarction (STEMI) increased each year [2]. According to Chinese Ministry of Health epidemiological survey data, the number of people who die from coronary heart disease each year in China is estimated at more than 1 million. Furthermore, the number of deaths of men aged 35–55 has increased rapidly [1]. Men aged <55 years and women <65 years with CHD are considered to have premature CHD [3]. With changing standards of living, dietary habits, modes of work, exercise levels, and genetic factors, the onset age for CHD has gradually decreased. Meanwhile, medical costs for young CHD patients have increased considerably. This study analyzed the clinical characteristics, risk factors, medical therapies, and prognoses of young (≤45 years) CHD patients hospitalized in two facilities in Beijing between 2010 and 2014. This study aims to provide further clinical data regarding secondary prevention and treatment of CHD.

## Methods

### Subjects

From January 2010 to December 2014, 5288 young (≤45 years) CHD patients were enrolled from the cardiac centers of Chinese PLA General Hospital and Anzhen Hospital, both in Beijing. The study protocol was approved by the Chinese PLA General Hospital and Anzhen Hospital Review Boards, and both informed consent and consent for publication were obtained from all participants.

- *Inclusion criteria* were: hospitalized in cardiac center during an established period; underwent coronary angiography; in accordance with the 1979 World Health Organization (WHO) standard, stenosis of left main artery ≥30%, or one or more of the left anterior descending branch (LAD), left circumflex branch (LCx), or right coronary artery (RCA) ≥50%; diagnosed with CHD.
- *Exclusion criteria* were: severe cardiomyopathy; rheumatic heart disease; congenital heart disease; severe congenital heart disease; malignant tumor; use of oral contraceptive pill or currently pregnant.

### Clinical data collection

All data were derived from the clinical data of the hospitalized patients. Risk factors analyzed included age, sex, smoking, drinking, hypertension, hyperlipidemia, diabetes, and family history of CHD.

Laboratory data were collected upon admission to the hospital, including levels of total cholesterol, triglycerides, low-density lipoprotein (LDL), high-density lipoprotein (HDL), hemoglobin, N-terminal pro-B-type natriuretic peptide (NT-proBNP), serum creatinine, troponin-T, creatine kinase-MB, urea nitrogen, and uric acid. Echocardiographic parameters were assessed by transthoracic echocardiography using the Teichholz method prior to coronary angiography. Parameters analyzed included thickness of the interventricular septum, left ventricular end-diastolic inner diameter, and left ventricular ejection fraction.

All medical treatments during hospitalization were recorded, including aspirin, clopidogrel, statin, ticagrelor, ACEI/ARB, β-blockers, nitrates, diuretics and digoxin.

### Follow-up

All subjects were followed up for 1 year after their first hospitalization. In the hospital, major adverse events recorded included cardiogenic shock, major bleeding, atrioventricular block (AVB), ventricular tachyarrhythmia (VT), ventricular fibrillation (VF) and thrombosis. After hospital discharge, major adverse events were defined, including acute myocardial infarction (MI), cardiac death, re-percutaneous coronary intervention (re-PCI), re-coronary artery bypass grafting (re-CABG), and stroke.

### Definition

The primary endpoints were death and complications during hospitalization. Complications during hospitalization included cardiogenic shock, VT or VF requiring anti-arrhythmic drugs or defibrillation, AVB requiring temporary cardiac pacemaker insertion, and major bleeding. The thrombolysis in myocardial Infarction (TIMI) bleeding criteria is used in this article [4]. Major bleeding was defined as including any intracranial bleeding (excluding microhemorrhages <10 mm evident only on gradient-echo magnetic resonance imaging), or clinically overt signs of hemorrhage associated with a drop in hemoglobin of ≥5 g/dL, or fatal bleeding (directly results in death within 7 days). The secondary endpoint was any major adverse cardiovascular events (MACE) during the follow-up period, including cardiac death, MI stroke, and emergency or elective repeat revascularization. Cardiac death was defined as mortality not resulting from noncardiac disease. If two or more complications occurred in a single patient, each complication type was recorded. After discharge, any MACEs during the follow-up period were recorded.

### Statistical analysis

Statistical analyses were performed using the Statistical Package for Social Sciences software (SPSS, version 18.0). Continuous variables with normal distributions were expressed as mean ± standard deviation, and compared using one-way analysis of variance. Categorical variables were compared using the chi-square test where appropriate. MACE was estimated by the unadjusted Kaplan–Meier method in the five groups from 2010 to 2014.

## Results

### Trends of clinical baseline data in young CHD patients

**Fig. 1:**
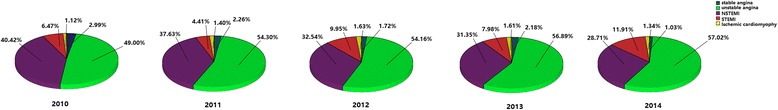
Composition changes of coronary heart disease during the past 5 years from 2010 to 2014. 1: stable angina; 2: unstable angina; 3: non-ST segment elevation myocardial infarction (NSTEMI); 4: ST segment elevation myocardial infarction (STEMI); 5: Ischemic cardiomyopathy. The rate of stable angina pectoris and STEMI increased, while the NSTEMI decreased (*P* < 0.05)

From 2010 to 2014, the average onset age of CHD young patients demonstrated no significant change. The rates of unstable angina and ST-segment elevation MI (STEMI) increased, while non-ST-segment elevation MI (NSTEMI) trended downward (see Table 1, Fig. 1). There was no significant change in mean blood pressure during hospitalization, but both heart rate and body mass index (BMI) slightly increased (72.8 ± 11.8 bpm vs. 74.3 ± 12.7 bpm; 27.9 ± 3.1 vs. 28.1 ± 3.1; *P* < 0.05, Table 1). The rates of various comorbidities, including hypertension, diabetes, and hyperlipidemia, presented upward trends over the 5 years, while smoking and alcohol consumption declined (Table 1). There were no differences among patients who from geographically different regions such cities or rural areas (*P* > 0.05). However, the proportion of those with high academic achievement trended upward (*P* < 0.01). The young CHD patients among all hospitalized patients, and such patients in the cardiac clinic among all patients in the cardiac clinic, trended significantly upward (5.4 to 7.2, and 1.2 to 1.7%, respectively, *P* < 0.01).

**Table 1:** Clinical characteristics of young CHD patients from 2010 to 2014

Variable	2010 (*n* = 804)	2011 (*n* = 930)	2012 (*n* = 1045)	2013 (*n* = 1241)	2014 (*n* = 1268)	*P*
Age	40.4 ± 4.2	40.6 ± 4.1	40.7 ± 4.2	40.8 ± 4.2	40.8 ± 4.2	NS
Male (%)	766 (91.2)	876 (94.2)	983 (94.1)	1146 (92.3)	1184 (93.4)	NS
SBP (mmHg)	123.9 ± 16.9	124.8 ± 16.9	123.9 ± 14.9	123.7 ± 15.3	123.5 ± 15.5	NS
DBP (mmHg)	78.2 ± 12.4	77.9 ± 12.1	77.0 ± 11.2	77.9 ± 11.4	77.6 ± 11.9	NS
HR	72.8 ± 11.8	72.4 ± 11.7	72.1 ± 11.2	74.3 ± 12.7	72.0 ± 11.0	0.000
BMI	27.9 ± 3.1	27.9 ± 3.2	27.7 ± 3.2	28.1 ± 3.1	27.6 ± 3.4	0.002
Risk factor						
Hypertension (%)	342 (40.7)	379 (40.7)	496 (47.5)	498 (40.1)	529 (41.7)	0.005
Diabetes (%)	171 (20.3)	182 (19.6)	238 (22.7)	324 (26.1)	284 (22.4)	0.006
Hyperlipidemia (%)	230 (27.3)	244 (26.2)	371 (35.5)	362 (29.1)	453 (35.7)	0.000
Smoke (%)	502 (59.7)	572 (61.5)	646 (61.8)	710 (57.2)	728 (57.4)	0.020
Alcohol use (%)	210 (25.0)	255 (27.4)	325 (31.1)	323 (26.0)	317 (25.0)	0.014
Geographical differences						
City area (%)	543 (67.5)	605 (65.1)	651 (65.2)	857 (69.1)	843 (66.5)	NS
Scholarship						0.000
High (%)	436 (51.9)	466 (50.1)	594 (56.8)	735 (59.2)	744 (58.7)	
Middle (%)	269 (32.0)	369 (39.7)	331 (31.7)	376 (30.3)	384 (30.3)	
Low (%)	99 (11.8)	95 (10.2)	120 (11.5)	130 (10.5)	140 (11.0)	
Diagnosis						0.000
SA	24 (2.8)	21 (2.2)	18 (1.7)	27 (2.1)	13 (1.0)	0.024
UA	394 (46.9)	505 (54.3)	566 (54.1)	706 (56.8)	723 (57.0)	0.003
NSTEMI	325 (38.7)	350 (37.6)	340 (32.5)	389 (31.3)	364 (28.7)	0.000
STEMI	52 (6.2)	41 (4.4)	104 (9.9)	99 (7.9)	151 (11.9)	0.000
ICM	9 (1.1)	13 (1.4)	17 (1.6)	20 (1.6)	17 (1.3)	NS
Proportion of young CAD the total hospitalized patients (%)	5.4	5.8	5.7	6.9	7.2	0.000
Proportion of young CAD in the total Cardiac clinic (%)	2147 (1.2)	2085 (1.1)	2283 (1.2)	3279 (1.5)	3732 (1.7)	0.000

*Abbreviations*: *SBP* systolic blood pressure, *DBP* diastolic blood pressure, *HR* heart rate, *BMI* body mass index, *SA* stable angina, *UA* unstable angina, *NSTEMI* non-ST-segment myocardial infarction, *STEMI* ST-segment elevation myocardial infarction, *ICM* ischemic cardiomyopathy, *NS* not significant

### Laboratory analysis and echocardiography

From 2010 to 2014, total cholesterol (4.54 ± 1.26 to 4.35 ± 1.26) and triglyceride levels (3.12 ± 6.26 to 2.49 ± 6.83) decreased (*P* < 0.05, Table 2). LDL cholesterol decreased slightly, without statistical significance (*P* > 0.05), and HDL cholesterol showed a significant decline (*P* < 0.05). Blood glucose levels and glycosylated hemoglobin showed no significant change (*P* > 0.05). The C-reactive protein (CRP) presented downward trends from 2010 to 2014 (5.22 ± 1.13 to 3.38 ± 0.98; *P* < 0.01). Left ventricular end diastolic diameter and ventricular septum thickness decreased (*P* < 0.05), while the ejection fraction had no significant change.

**Table 2:** Biochemical parameters and echocardiogram findings

Variable	2010 (*n* = 804)	2011 (*n* = 930)	2012 (*n* = 1045)	2013 (*n* = 1241)	2014 (*n* = 1268)	*P*
Biochemical						
TC (mmol/L)	4.54 ± 1.26	4.48 ± 1.16	4.44 ± 1.35	4.42 ± 1.19	4.35 ± 1.26	0.020
TG (mmol/L)	3.12 ± 6.26	2.23 ± 1.51	2.28 ± 1.88	2.31 ± 1.89	2.49 ± 6.83	0.026
LDL (mmol/L)	3.21 ± 8.39	2.26 ± 0.96	2.75 ± 1.09	2.74 ± 1.01	2.77 ± 3.26	NS
HDL (mmol/L)	0.99 ± 0.22	0.94 ± 0.23	0.91 ± 0.21	0.89 ± 0.21	0.89 ± 0.20	0.000
Creatinine (mg/dL)	82.59 ± 34.06	78.41 ± 16.91	78.83 ± 47.32	77.85 ± 45.99	77.79 ± 18.38	0.030
BUN (mmol/L)	7.61 ± 4.85	7.29 ± 4.55	7.66 ± 5.17	7.51 ± 4.54	7.18 ± 4.53	NS
UA (μmol/L)	372.1 ± 111.2	355.5 ± 88.9	359.9 ± 91.3	367.9 ± 93.4	380.4 ± 91.9	0.000
ALT (U/L)	50.2 ± 48.3	45.4 ± 35.2	48.6 ± 43.1	46.8 ± 41.4	48.1 ± 42.0	NS
AST (U/L)	62.3 ± 92.7	56.7 ± 89.5	52.2 ± 77.2	51.2 ± 74.3	53.1 ± 77.4	NS
CK (U/L)	386.9 ± 875.3	368.3 ± 894.6	314.4 ± 737.5	336.8 ± 718.7	377.7 ± 928.1	NS
CKMB (ng/ml)	33.7 ± 76.1	29.4 ± 82.1	20.8 ± 60.5	26.0 ± 138.1	29.5 ± 141.3	NS
cTnT (ng/ml)	1.94 ± 1.43	1.82 ± 1.31	1.87 ± 1.53	1.97 ± 2.23	1.95 ± 1.97	NS
NT-proBNP (pg/ml)	657.0 ± 3973.8	385.6 ± 1348.7	372.8 ± 1638.5	206.8 ± 572.4	197.0 ± 561.7	0.000
Glucose (mmol/L)	6.73 ± 2.89	6.45 ± 2.51	6.57 ± 2.71	6.67 ± 2.68	6.56 ± 2.88	NS
HbA1c (%)	6.86 ± 1.76	6.64 ± 1.72	6.71 ± 1.71	6.16 ± 1.70	6.36 ± 1.63	NS
CRP (mg/dl)	5.22 ± 1.13	4.28 ± 1.28	4.43 ± 1.56	3.54 ± 1.25	3.38 ± 0.98	0.000
Hematologic						
Hemoglobin (g/L)	147.4 ± 16.2	147.5 ± 15.3	146.2 ± 15.6	146.8 ± 16.0	147.6 ± 15.0	NS
Red cell count (10*^12^/L)	5.90 ± 1.89	5.91 ± 1.92	6.00 ± 1.91	6.15 ± 1.94	6.09 ± 1.99	0.017
White cell count (10*9/L)	9.20 ± 3.69	8.57 ± 2.99	8.41 ± 3.08	8.59 ± 3.34	8.74 ± 3.36	0.005
Platelet count	230.9 ± 72.7	228.8 ± 5.96	225.6 ± 58.8	233.8 ± 58.2	236.1 ± 64.2	NS
RDW (%)	12.96 ± 0.69	13.03 ± 0.62	12.80 ± 0.71	12.71 ± 0.79	12.91 ± 1.28	0.018
Echocardiography						
LVDd (mm)	50.28 ± 5.38	49.64 ± 5.27	49.87 ± 5.84	49.92 ± 5.71	49.42 ± 5.44	0.028
IVST (mm)	10.07 ± 1.53	10.21 ± 1.51	10.13 ± 1.59	10.32 ± 1.58	10.05 ± 1.60	0.002
EF (%)	59.3 ± 9.2	59.8 ± 8.9	59.8 ± 9.5	59.8 ± 9.1	59.8 ± 9.0	NS

*Abbreviations*: *TC* total cholesterol, *TG* triglyeride, *LDL* low density lipoprotein cholesterol, *HDL* high density lipoprotein cholesterol, *Cr* creatinine, *BUN* blood urea nitrogen, *UA* uric acid, *ALT* alanine aminotransferase, *AST* Aspartate transaminase, *CK* creatine kinase, *CKMB* creatine kinase MB isoenzyme, *cTnT* cardiac Troponin T, *NT-proBNP* N-terminal pro-B-type natriuretic peptide, *HbA1c* hemoglobin A1c, *LVDd* left ventricular end-diastolic dimension, *IVST* interventricular septal thickness, *EF* ejection fraction

### Analysis of coronary artery lesion

Analysis of coronary angiography in young CHD patients frequently showed single vessel lesions, specifically of the LAD, RCA, LCx, and left main artery. From 2010 to 2014, there were no significant changes in the composition of these lesions, except that the proportion of left main artery lesions slightly increased (*P* < 0.05, Table 3). In accordance with ACC/AHA classification of coronary heart disease, our statistical analysis showed a decreasing trend of A lesions, and an increasing trend of C lesions. There was no significant change in TIMI flow classification.

**Table 3:** Baseline coronary angiographic findings

Variable	2010 (*n* = 804)	2011 (*n* = 930)	2012 (*n* = 1045)	2013 (*n* = 1241)	2014 (*n* = 1268)	*P*
Involved vessel number (%)						
1 Branch						
LAD (%)	256 (31.8)	320 (34.4)	368 (35.2)	408 (32.9)	384 (30.3)	NS
LCx (%)	54 (6.7)	56 (6.0)	59 (5.6)	70 (5.6)	85 (6.7)	NS
RCA (%)	85 (10.5)	100 (10.7)	115 (11.0)	143 (11.5)	134 (10.6)	NS
2 branch (%)	235 (29.2)	292 (31.4)	288 (27.6)	365 (29.4)	387 (30.5)	NS
3 branch (%)	160 (19.9)	157 (16.9)	211 (20.2)	241 (19.4)	268 (21.1)	NS
LM (%)	14 (1.7)	5 (0.5)	4 (0.38)	14 (1.1)	10 (0.8)	0.018
ACC / AHA classification						
A (%)	455 (56.6)	542 (58.3)	597 (57.1)	672 (54.1)	654 (51.6)	0.011
B1 (%)	95 (11.8)	103 (11.1)	129 (12.3)	149 (12.2)	133 (10.5)	NS
B2 (%)	112 (13.9)	144 (15.5)	148 (14.2)	202 (16.3)	225 (17.7)	NS
C (%)	142 (17.7)	141 (15.1)	171 (16.4)	218 (17.6)	256 (20.2)	0.029
TIMI flow (%)						
TIMI 0	218 (27.1)	223 (24.0)	266 (25.5)	347 (28.0)	368 (29.0)	NS
TIMI 1	2 (0.2)	12 (1.3)	3 (0.3)	4 (0.3)	6 (0.5)	0.007
TIMI 2	16 (2.0)	13 (1.4)	6 (0.6)	22 (1.8)	14 (1.1)	NS
TIMI 3	568 (70.6)	682 (73.3)	770 (73.7)	868 (69.9)	880 (69.4)	NS

*Abbreviations*: *LAD* left anterior descending artery, *LCx* left circumflex, *RCA* right coronary artery, *NS* not significant

### Medical treatment

**Fig. 2:**
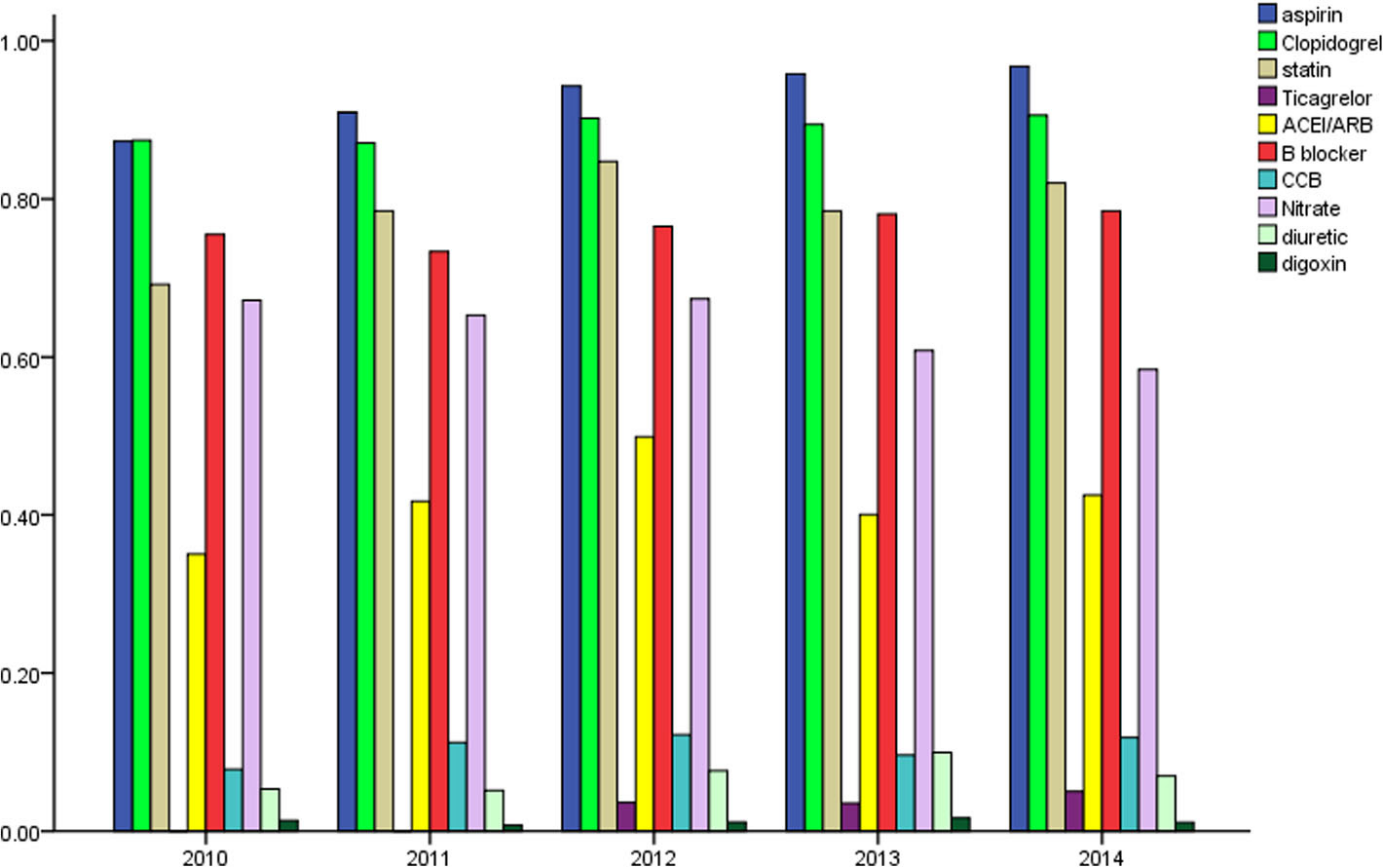
Change of medical use during hospitalization in2010–2014. Usage of aspirin, clopidogrel, ticagrelor, statins, and ß-blockers increased overall (*P* < 0.05)

Regarding anti-platelet treatment, the use of aspirin increased from 87.3 to 96.7% over the 5 years, clopidogrel use had a slight increase, and ticagrelor use increased from 3.6 to 5.1% (*P* < 0.05, Fig. 2). Use of statins also increased: from 69.1 in 2010, to 84.7 in 2012, to 82.1% in 2014 (*P* < 0.05). Use ofβ-blockers was around 75% with no significant change. ACEI/ARB also increased: from 35.1% in 2010, to 49.8% in 2012, to 42.5% in 2014 (*P* < 0.05). Conversely, use of nitrates decreased slightly, from 67.1 to 58.4% (*P* < 0.05). Use of other medical treatments, such as calcium channel blockers (CCB), diuretics, and digoxin, ranged around lower levels.

### Events in hospital and follow-up

The rates of death (from 1.8 to 0.6%) and cardiac shock (from 2.4 to 0.4%) in the hospitals decreased (*P* < 0.05, Table 4), while the rates of AVB, VT, VF, and major bleeding had no significant change. During the 1-year follow-up, incidences of cardiac death, MI, PCI, CABG, and stroke did not significantly change, nor did total MACE events.

**Table 4:** In- and out-hospital outcomes from 2010 to 2014

	2010	2011	2012	2013	2014	*P*
In-hospital outcome	(*n* = 804)	(*n* = 930)	(*n* = 1045)	(*n* = 1241)	(*n* = 1268)	
Death (%)	15 (1.8)	10 (1.1)	3 (0.3)	2 (0.2)	7 (0.6)	0.000
Complications (%)						
Cardiogenic shock	17 (2.1)	19 (2.0)	4 (0.4)	8 (0.6)	5 (0.4)	0.000
Major bleeding	8 (1.0)	7 (0.8)	12 (1.1)	10 (0.8)	12 (0.9)	NS
AV block	2 (0.2)	1 (0.1)	2 (0.2)	3 (0.2)	-	NS
VT and/or VF	22 (2.7)	25 (2.7)	22 (21.1)	21 (16.9)	23 (18.1)	NS
Thrombosis	2 (0.2)	4 (0.4)	3 (0.3)	7 (0.6)	7 (0.6)	NS
Out-hospital outcome	*N* = 738	*N* = 840	*N* = 1017	*N* = 1216	*N* = 1257	
MACE (%)	18 (2.4)	18 (2.1)	22 (2.2)	17 (1.4)	20 (1.6)	NS
Cardiac death	1 (0.1)	-	1 (0.1)	3 (0.2)	1 (0.1)	NS
MI	10 (1.3)	14 (1.7)	14 (1.4)	13 (1.1)	16 (1.3)	NS
Re-PCI	17 (2.3)	18 (2.1)	22 (2.2)	17 (1.4)	19 (1.5)	NS
CABG	1 (0.1)	-	-	-	1 (0.1)	NS
Stroke	-	-	-	1	-	NS

*Abbreviations*: *VT* ventricular tachycardia, *VF* ventricular fibrillation, *MACE* major adverse cardiac event, *PCI* percutaneous coronary intervention, *TVR* target vessel revascularization, *TLR* target lesion revascularization, *MI* myocardial infarction, *CABG* coronary artery bypass graft

## Discussion

The prevalence of cardiovascular disease in China is increasing, an estimated 2.9 billion people with cardiovascular disease in China [1], 2.7 billion people with hypertension, 7 million with stroke, 2.5 million with MI, and 4.5 million with heart failure. Li et al. reported that during the past decade in China, hospital admissions for STEMI have risen; in these patients, comorbidities and the intensity of testing and treatment have also increased [2]. Quality of care has improved for some treatments, but vital gaps persist, and in the hospital, mortality has not decreased. National efforts are needed to improve care and outcomes of STEMI patients in China. Premature coronary heart disease is defined as men with onset age <55 years, and women with onset age <65 years. The present study showed that from 2010 to 2014 in China, the proportion of young CHD patients in the department of cardiology, as well as the proportion of young CHD outpatient in the total outpatient cardiac clinic department trended upward, which may be partly associated with the rising incidence of young patients. With changing lifestyles, diets, modes of working, and genetic factors, the onset age of CHD has gradually gone down, as young CHD patients with onset age <45 gradually increase. The present study found that BMI of young CHD patients had an increasing trend; BMI was 27.6 at the upper limit, and the proportion of young obese people was greater [5]. Moreover, incidence of CHD risk factors, including hypertension, diabetes, and hyperlipidemia, increased. The proportion of smoking among young people has declined, but the overall proportion maintains a high ratio. Therefore, life-style changes, rehabilitation exercise, weight control, stopping smoking, and reducing hypertension, diabetes, and hyperlipidemia, are all key objectives for secondary prevention of young CHD.

The present study found that from 2010 to 2014, the number of young CHD patients has shown an upward trend, especiallyamong men. The rates of unstable angina and STEMI increased significantly, consistent with the ChinaPEACE results, while NSTEMI decreased. Further analysis and comparison showed that the average blood pressure of young CHD patients during hospitalization did not change significantly.

Previous studies [6] have shown that heart rate control was associated with angina symptoms and prognosis. This study showed that average heart rate remains high during hospitalization, with an average of ≥72 bmp. The use of ß-blockers increased over this 5-year period, but overall use was <80%. The incidence of hypertension, diabetes mellitus, and hyperlipidemia also rose. A report on cardiovascular disease in China in 2014 showed that the prevalence of hypertension had significantly increased [1]. In 2002, a survey showed that 18% of people in China aged >18 years had hypertension. Those with hypertension had increased to 270 million as of 2014 [1]. In the present study, the prevalence of hypertension in young CHD patients from 2010 to 2014 increased from 40.7 to 47.5%, with a slight downward trend. Diabetes is also an important risk factor in cardiovascular disease [7]. A 1999 survey showed that the prevalence of diabetes in China was about 9%, and increased to 11.6% as of 2010 [1]. The overall proportion of those with acute coronary syndrome (ACS) in youth patients (including unstable angina, STEMI and NSTEMI) increased; with an especially notable rises in the proportion of unstable angina. This may be associated with increased risk factors for young CHD, which underscores the need for secondary prevention.

The present study shows that from 2010 to 2014 fasting blood glucose levels and glycosylated hemoglobin declined slightly without statistical difference, and the incidence of diabetes increased from 20.3 to 26.1%; far from optimistic figures. Hyperlipidemia increased significantly; results from national research in 2010 showed that total cholesterol levels were ≥6.22 mmol/L for men and women, with prevalence of 3.2 and 3.4%, respectively. The results of the present study showed that from 2010 to 2014, the incidence of hyperlipidemia in young CHD patients increased from 27.3 to 35.7%, while the percentage of patients receiving statin therapy is still <80%. This study also found that BMI shows an increasing trend, while obesity and being overweight are also potential risk factors for CHD. Here we also found that CRP decreased from 2010 to 2014, which may be associated with a decrease of the proportion of myocardial infarction as shown in another report [8].

The report of cardiovascular disease in China in 2014 shows that the proportion of overweight urban and rural residents is about 30%, with obesity is at 12%. These are potentially susceptible populations for CHD. BMI showed a significantly increasing trend in young CHD patients. Obesity can promote proliferation of vascular endothelial cells and activation of inflammatory response, which are associated with endothelial dysfunction and atherosclerosis [9–11]. Obesity can also lead to accelerated vascular aging, and increased risk of atherosclerotic disease [12–15]. Smoking is associated with blood lipid disturbance, inflammation, and vascular endothelial dysfunction [16–18]. Smoking has been considered one of the main risk factors for CHD among young people [19]. The present study further showed that, despite the declines in smoking and drinking, the overall proportions for both are still high, and the average incidences exceed 50 and 25%, respectively. Analysis of laboratory test results showed that total cholesterol and triglyceride levels showed a downward trend, while LDL cholesterol did not change significantly. This is associated with lack of exercise, obesity, and other lifestyle choices, as also shown in both the Coronary Artery Risk Development in Young Adults Study and Cardiovascular Risk in Young Finns Study [20, 21]. A large number of studies proved statins could reduce the incidence of cardiovascular events and inhibit development of coronary plaque [22]. However, the present study showed that in the hospital, <80% of young CHD patients received statin therapy. We also found the proportion presented with upstream trend, which was partly due to lack of exercise.

The olderly patients presented with multiple, diffuse, and calcification lesions, while young CHD patients presented with acute onset, less collateral circulation, and more total occlusive disease [23]. The present study showed that young CHD patients presented with single branch lesions, mainly in the LAD, followed by RCA, and LCx. As per ACC/AHA classification [24], this study showed the incidence of simple type A lesions had reduced, while the complex type C lesions increased over the course of the examined years. The events in-hospital, overall mortality, and cardiogenic shock trended downward trend, and major bleeding, malignant ventricular arrhythmia, and acute or subacute thrombus demonstrated no difference. This may be partly associated with improvements in medical and interventional treatments. No significant improvement in MACE, including cardiac death, myocardial infarction, CABG, PCI, and stroke incidence, was found after 1 year.

## Conclusions

Ultimately, this study confirmed that from 2010 to 2014, with updated guidelines applied in clinical treatment, secondary prevention has gradually been promoted. However, many shortcomings are still seen, such as the rising incidence of hypertension, diabetes, hyperlipidemia, and higher BMI. The ratio of smokers is still high. In medical treatment, use of aspirin, clopidogrel, statins, and ß-blockers have improved overall, but the control of heart rate and blood lipids levels are still low. A great deal of progress is still needed for young patients regarding primary and secondary prevention of CHD, including BMI control, smoking cessation, rehabilitation exercise, and reducing hypertension, diabetes and hyperlipidemia.

## Additional files

Additional file 1: Clinical data of young CHD patients from 2010 to 2014. (JPG 405 kb)
Additional file 2: Ethical approval for this study. (SAV 209 kb)

## Abbreviations

ACEI: Angiotensin converting enzyme inhibitors
AMI: Acute myocardial infarction
ARB: Angiotensin receptor blocker
AVB: Atrioventricular block
CHD: Coronary heart disease
HDL: High-density lipoprotein
LAD: The left anterior descending branch
LCx: Left circumflex branch
LDL: Low-density lipoprotein
MACE: Major adverse cardiovascular events
NT-proBNP: N-terminal pro-B-type natriuretic peptide
RCA: Right coronary artery
re-CABG: Re-coronary artery bypass grafting
re-PCI: Re-percutaneous coronary intervention
STEMI: ST-segment elevation myocardial infarction
TIMI: Thrombolysis in myocardial infarction
VF: Ventricular fibrillation
VT: Ventricular tachyarrhythmia
