# Supplementary figures and images for: Trend in young coronary artery disease in China from 2010 to 2014: a retrospective study of young patients ≤ 45
Source: BMC Cardiovasc Disord. 2017 Jan 7;17:18. doi: 10.1186/s12872-016-0458-1 (PMC5219759; doi:10.1186/s12872-016-0458-1)

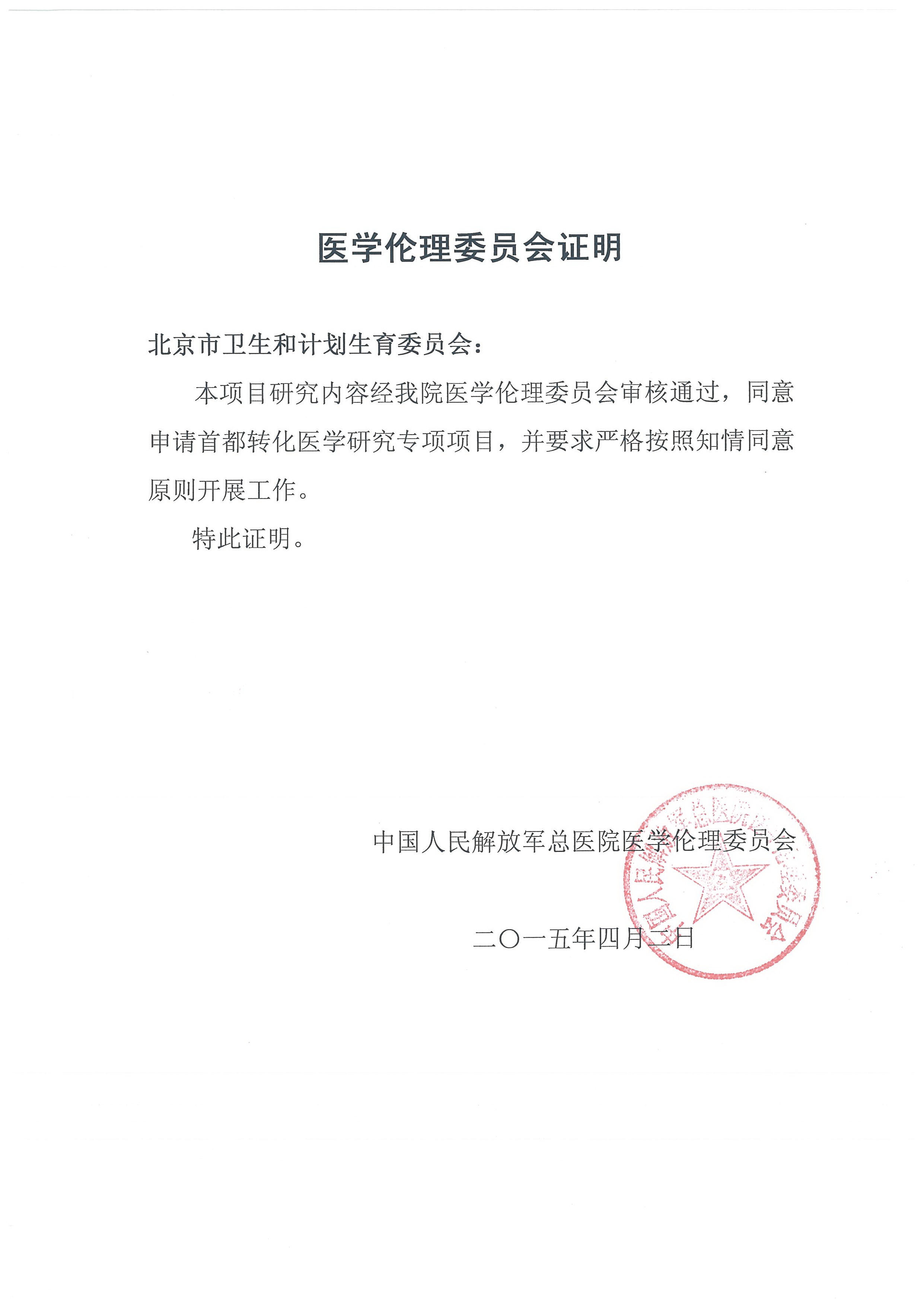

Supplement: Additional file 1: — Clinical data of young CHD patients from 2010 to 2014. (JPG 405 kb) [file 12872_2016_458_MOESM1_ESM.jpg]
